# MTRAP: Pairwise sequence alignment algorithm by a new measure based on transition probability between two consecutive pairs of residues

**DOI:** 10.1186/1471-2105-11-235

**Published:** 2010-05-08

**Authors:** Toshihide Hara, Keiko Sato, Masanori Ohya

**Affiliations:** 1 Department of Information Sciences, Tokyo University of Science, 2641 Yamazaki, Noda City, Chiba, Japan

## Abstract

**Background:**

Sequence alignment is one of the most important techniques to analyze biological systems. It is also true that the alignment is not complete and we have to develop it to look for more accurate method. In particular, an alignment for homologous sequences with low sequence similarity is not in satisfactory level. Usual methods for aligning protein sequences in recent years use a measure empirically determined. As an example, a measure is usually defined by a combination of two quantities (1) and (2) below: (1) the sum of substitutions between two residue segments, (2) the sum of gap penalties in insertion/deletion region. Such a measure is determined on the assumption that there is no an intersite correlation on the sequences. In this paper, we improve the alignment by taking the correlation of consecutive residues.

**Results:**

We introduced a new method of alignment, called MTRAP by introducing a metric defined on compound systems of two sequences. In the benchmark tests by PREFAB 4.0 and HOMSTRAD, our pairwise alignment method gives higher accuracy than other methods such as ClustalW2, TCoffee, MAFFT. Especially for the sequences with sequence identity less than 15%, our method improves the alignment accuracy significantly. Moreover, we also showed that our algorithm works well together with a consistency-based progressive multiple alignment by modifying the TCoffee to use our measure.

**Conclusions:**

We indicated that our method leads to a significant increase in alignment accuracy compared with other methods. Our improvement is especially clear in low identity range of sequences. The source code is available at our web page, whose address is found in the section "Availability and requirements".

## Background

Under a rapid increase of genome data, the need for accurate sequence alignment algorithms has become more and more important, and several methods have been developed. Sequence alignment algorithm is designed for mainly two purposes. One purpose is to design for comparing a query sequence with the database which contains preobtained sequences, and another is to design for generating multiple sequence alignment. FASTA [1] and BLAST [2], commonly used methods in molecular biology, are developed for database search, where a quick alignment algorithm is desired. For this quickness, the accuracy of alignment in these methods is lower than of the alignment by optimal algorithm.

In addition to for database search, sequence alignment is used for generating multiple alignment. In the multiple alignment, the accuracy is more important than the quickness. The recent popular multiple alignment methods, such as ClustalW [3], DIALIGN [4], TCoffee [5], MAFFT [6], MUSCLE [7], Probcons [8] and Probalign [9], are based on a "pairwise" alignment algorithm. In order to generate alignment with a realistic time and space costs, all of these methods use a progressive algorithm for constructing multiple alignment [10]. This "progressive" means to construct the multiple alignment by iterating pairwise alignment. These kind of methods give high accuracy for closely-related homologous sequences with identity more than 40%, but are not satisfied for distantly-related homologous sequences [11]. To improve the accuracy of the progressive algorithm, some measures based on, for instance, entropy [12] or consistency [13] have been developed. However, these measures are still not taken the intersite correlations of the sequences.

According to Anfinsen's dogma (also known as the thermodynamic hypothesis) [14], for a small globular protein, its three-dimensional structure is determined by the amino acid sequence of the protein. There may exist intersite correlations at least for two consecutive pairs of residues. Gonnet et al. considered this possibility [15]. We could improve alignment accuracy by taking into account information of the intersite correlations. Recently, Crooks et al. tried and tested such an approach [16], but they concluded that their approach is statistically indistinguishable from the standard algorithm. More recently, however, Lu and Sze proposed another approach [17], and they concluded that their strategy is able to consistently improve over existing algorithms on a few sets of benchmark alignments. Their approach is a kind of post processing algorithm. They take the average of the optimal values of the neighboring sites of one site, and they consider that the average value is the optimal value of that site. Note that they used usual "sum of pairs" measure for sequences. Their improvement of the accuracy in their tests was around 1~ 3% by using the BAliBASE 3.0 [18].

In this paper, we propose another approach introducing a new metric defined on compound systems of two sequences. Most of alignments are based on finding a path that gives the minimum value to the sum of difference (the maximum value to the sum of similarity) for each residue pair between two sequences. Our method is to change the way defining the difference (so, the sum) above by computing this sum of differences by introducing a quantity through the transition probability between consecutive pairs of residues. The comparison of our method with the method of Lu and Sze gives the following: In the very difficult range that the sequence identity is less than 15%, our method improves the accuracy nearly 8% up, but the Lu and Sze method improves it nearly 1% up.

### A new measure taking the correlation of consecutive pairs of residues

First, let us establish some notations. Let Ω be the set of all amino acids, and Ω* be the Ω with the indel (gap) "*": Ω* ≡ Ω∪{*}. Let [Ω*] be the set of all sequences of the elements in Ω*. We call an element of Ω a residue and an element of Ω* a symbol. In addition, let Γ ≡ Ω × Ω be the direct product of two Ωs and Γ* ≡ Ω* × Ω*.

Consider two arranged sequences, *A *= *a*_1_*a*_2 _... *a*_*n *_and *B *= *b*_1_*b*_2 _... *b*_*n*_, both of length *n*, where *a*_*i*_, *b*_*j *_∊ Ω*. We also denote the sequences by *u*_1_*u*_2 _... *u*_*n*_, where *u*_*i *_= (*a*_*i*_, *b*_*i*_) ∊ Γ*, and we call *u*_*i *_a site in the following discussion. In general, the relative likelihood that the sequences are related as opposed to being unrelated is known as the "odds ratio":(1)

Here, *p *(*a*) is the occurrence probability of the given segment and *p *(*a*; *b*) is the joint probability that the two segments occur. In order to arrive at an additive scoring system, Equation (1) is typically simplified by assuming that the substitutions are independent of the location and there is no intersite correlations; namely, *p *(*A*) = ∏ *p *(*a*), *p *(*B*) = ∏ *p *(*b*) and *p *(*A, B*) = ∏ *p *(*a, b*). Thus the logarithm of Equation (1), known as the log-odds ratio, is now a sum of independent parts:(2)

Where(3)

is the log likelihood ratio of the symbol pair (*a, b*) occurring as an aligned pair to that occurring as an unaligned pair. The *s *(*a, b*) is called a score and S = (*s *(*a, b*)) is called a substitution matrix. These quantities (Equation (2) and (3)) are used to define a measure for pairwise sequence alignment [19]. Here, we define a normalized substitution matrix (i.e., every element in S takes the value between 0 and 1) and define a difference of *A *and *B*.

Let *f*_*s *_: [*s*_min, _*s*_max _] ↦ R be a normalizing function:(4)

Where(5)

Let put  (*a, b*) = *f *(*s *(*a, b*)) for *a, b *Ω. This  (*a, b*) is a normalized expression of the score *s *(*a, b*). By using this quantity, we define a normalized substitution matrix as M = ( (*a, b*)). Then a difference of *A *and *B *is defined by(7)

When the sequence *A *is equal to *B *the difference *d*_*sub *_(*A, B*) has a minimum value 0.

One of the essential assumption for the above approach (using a sum of independent parts as a difference of *A *and *B*) was the induction of the occurring probability. We could take more informative approach by including the intersite correlations. Crooks et al. tried one of such an approach [16]. They introduced a measure for two sequences based on a multivariate probability approximated by using the intersite relative likelihood. But, they concluded that their approach is statistically indistinguishable from the standard algorithm. We feel that their measure (equation (4) in their paper) is different from ours. To introduce their measure, they defined a type of joint probability. However it can not be a probability, bacause their quantity is the multiplication of likelihood "ratios", so it goes beyond more than 1. Moreover, we think that the intersite relative likelihood may not describe the difference of sequence *A *and *B*. Under an assumption that each site of the sequences has Markov property, we propose a new measure for two sequences by adding a transition effect and its weight *ε *(a degree of mixture):(8)

where(9)

Here we introduce a normalized transition (*u*_*i*_, *u*_*i*+1_) called "Transition Quantity", in order to simplify the equation. Let (*u*_*i*_, *u*_*i*+1_) be a normalized transition defined as(10)

where *f*_*t *_(*x; u*) is a normalizing function:(12)

By using the above quantity, a difference of *A *and *B *representing the "intersite transition" is defined as(13)

Consequently, we define a difference measure for two sequences by combination of two differences *d*_sub _and *d*_trans_:(14)

### Estimation of the Transition Quantity

Let us discuss how to estimate the transition quantity . We can estimate the transition quantity by collecting reliable aligned protein sequences. In this study, we estimated the transition quantity by means of the superfamilies subset of the dataset SABmark (version 1.63) [20]. This set covers the entire known fold space using only high-quality structures taken from the SCOP database [21]. For a large set, the same sequences are re-used in the set. In order to reduce the bias introduced by multiple use of the same sequences, we assign a weight to each sequence. This approach is similar to the one described in the paper [22]. If a sequence occurs *N *times in the dataset, its weight is *N*^-1/2^. We estimated the transition quantity from the weighted frequencies of observed transitions as follows.

Let ^*L*, *N *^be the set of *N *sequences with length *L*:

Let  = (*a*_*ik*_, *a*_*jk*_) be in the finite set Γ* and it is a symbol pair in the *k*th site of the ^*L*, *N*^. In addition, let  be the set of all given sets ^*L*, *N *^(i.e., the superfamilies set of the SABmark), and let *N*_*A *_be the frequency of the sequence *A *in the set .

Let : Γ* × Γ* ↦ **R **be a mapping which represents the weighted frequency appearing the symbols (*u, v*) in ^*L*, *N *^such that(15)

Let *p *(*v\u*) be a transition probability from the symbol pair *u *to the pair *v *on  such that(17)

for *u, v *in the finite set Γ*.

We define a matrix T = ((*u,v*)) called "Transition matrix" by the elements (*u, v*) as

where *t *(*u, v*) = log *p *(*v\u*) and *f*_*t *_is the normalizing function defined by the equation (12).

### MTRAP Algorithm

**Figure 1:**
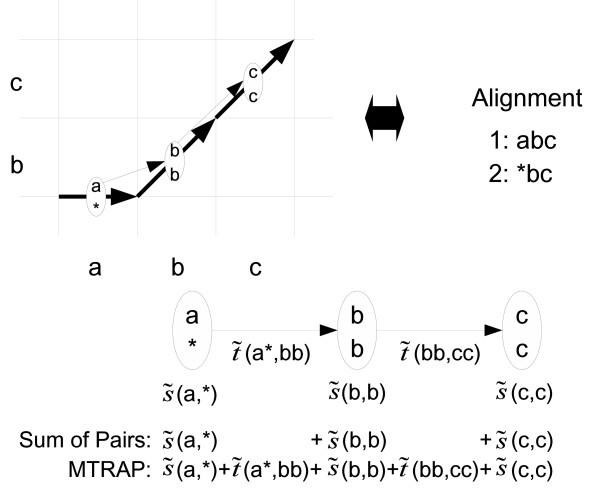
**Illustration of MTRAP**. The set of bold arrows indicates a possible route for alignment and the normal arrows indicate the transitions from a pair to the subsequent pair. A value (*u*) indicates a difference of two residues in a given pair *u *and (*u*_1_, *u*_2_) indicates the quantity based on a transition probability from a pair *u*_1 _to the subsequent pair *u*_2_. A conventional objective function such as "Sum of Pairs" is defined only by the sum of the value (*u*) of all possible pairs across the sequences, whereas our objective function in the MTRAP is defined by the sum of both (*u*) and (*u*_1_, *u*_2_) across the sequences (Equation (14)).

The MTRAP (sequence alignment method by a new Measure based on TRAnsition Probability) is an alignment algorithm by minimizing the value of a certain objective function based on the transition quantity (Figure 1). We describe the algorithm by means of dynamic programming [23].

**Figure 2:**
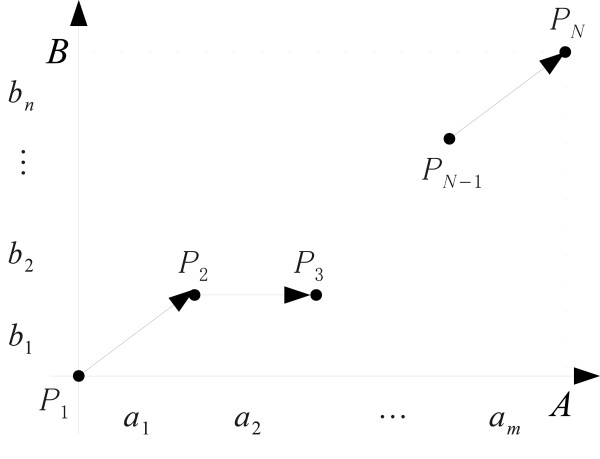
**Lattice points with two-sequences**. The input amino acid sequences *A *= (*a*_1 _⋯ *a*_*m*_) and *B *= (*b*_1 _⋯ *b*_*n*_) are placed on each two axes. An initial point *P*_1 _(0,0) and a final point *P*_*N *_(*m*, *n*) are fixed.

Let *A, B *be two amino acid sequences such as

where *a*_*i*_, *b*_*j *_∊ Ω. Take the lattice point *P*_*k *_= (*i*_*k*_, *j*_*k*_), *i *= 1, ..., *m*, *j *= 1, ..., *n *as in Figure 2. We call the sequence of the lattice points(18)

a "route" with an initial point *P*_1 _= (0, 0) and a final point *P*_*N *_= (*m, n*) if the following conditions are met:(19)

Let *α*_*R*_, *β*_*R *_be maps from a route ℛ = {*P*_1_, *P*_2_, ..., *P*_*N*_} to a set Ω* such that(20)

and *μ*_*R *_be a map from the route ℛ to the set of all symbol pairs Γ*(≡ Ω* × Ω*) such that(22)

We call the following A* and B* the alignment of *A *and *B *by the route ℛ:

Let *R*(*P*) be the set of all routes with the final point *P*, that is,(23)

Let us fix the following notations for the following discussion: (1) Γ*^- ^≡ Ω* × Ω, (2) Γ;^-^* ≡ Ω × Ω*, (3) Γ^*g*-^≡ {*} × Ω, (4) Γ^-*g *^≡ Ω × {*}, (5) *w*_open _is a constant called gap "opening" cost; 0 ≤ *w*_open _≤ 1, (6) *w*_extend _is a constant called gap "extending" cost; 0 ≤ *w*_extend _≤ *w*_open _and (7) *ε *is a weight, 0 ≤ *ε *≤ 1 (i.e., the mixture of usual difference *d*_sub _and our new difference *d*_trans_). The difference between *A *and *B *by a route ℛ is given by(24)

where *d*_*s *_is a function from Γ* × Γ* to **R **such that(25)

The degree of difference between *A *and *B *with respect to a final point *P *can be defined as(28)

Hence the degree of difference between *A *and *B *is(29)

We calculate *D*_*AB *_by a dynamic programming technique as below. For a final point *P*_*k *_= (*i, j*) and a route ℛ = {*P*_1_, ..., *P*_*k*_} ∊ *R*(*P*_*k*_), we have(30)

where *Q*_1 _= (*i *- 1, *j*), *Q*_2 _= (*i *-1, *j *- 1) and *Q*_3 _= (*i, j -1*). Therefore(31)

with(32)

for *l *= 1, 2, 3. Thus we obtain(33)

where(37)

for *l *= 1, 2, 3.

Each point *Q*_*l *_has three points  which possibly go to *Q*_*l *_one step after. These points are precisely written as(38)

when *Q*_1 _= (*i *-1, *j*), *Q*_2 _= (*i *-1, j -1), *Q*_3 _= (*i*, *j *- 1).

The distances *D*_*l *_(*P*_*k *_= (*i*, *j*)) can be obtained from one step before by the following recursion relations:(39)

for *l *= 1, 2, 3. The values *D*_*l *_of initial point and those of the edge points are assumed as(42)

Moreover for other special cases, the recursive relation of the edge points satisfies(46)

for ℛ ∊ *R*_1 _(*P*_*k*_), *i *= 1, ..., *m*,(47)

for ℛ ∊ *R*_3 _(*P*_*k*_), *j *= 1, ..., *n*.

This calculation is completed in *mn *steps.

### Multiple sequence alignment by MTRAP

In order to discuss the effect of using MTRAP algorithm in the iteration step of progressive multiple alignment, we modified the TCoffee [5], a consistency-based progressive multiple alignment program, by means of our distance (Equation (14)). TCoffee constructs a primary library (pairwise alignments between all of the sequences to be aligned) at first step. We implemented our algorithm to make this primary library. That is, our modified TCoffee constructs a multiple alignment by following steps.

1. Generating a primary library by using MTRAP

2. Extending the library (Calculate a consistency)

3. Making a guide tree for the progressive step

4. Constructing a multiple alignment by progressive strategy

The modified TCoffee uses the extended library obtained by the MTRAP algorithm for aligning.

### Performance evaluation

We compared the performance of MTRAP to those of the most often used nine methods: Needle, ClustalW2, MAFFT, TCoffee, DIALIGN, MUSCLE, Probcons, Probalign and TCoffee-Lu/Sze. The details of these nine methods are: (1) Needle, a global pairwise alignment using Needleman-Wunsch algorithm [24] contained in EMBOSS package ver. 5.0.0 [25]; (2) ClustalW2 [3,26], a typical progressive multiple alignment method; (3) MAFFT ver. 6 [6], a fast method with Fourier transform algorithm; (4) TCoffee ver. 5.31 [5], a heuristic consistency-based method that combines global and local alignments; (5)

DIALIGN ver. 2.2 [4], a method with segment-segment approach; (6) MUSCLE ver. 3.7 [7], a method with Log-Expectation algorithm; (7) Probcons ver. 1.12 [8], a probabilistic consistency-based method, (8) Probalign ver. 1.1 [9], a multiple sequence alignment using partition function posterior probabilities and (9) TCoffee-Lu/Sze, an improved TCoffee modified by the Lu/Sze algorithm [17]. These programs without MAFFT used their default parameters and MAFFT used "L-IN-i" strategy mode.

To measure the accuracy of each method, we used three different databases: HOMSTRAD (version November 1, 2008) [27,28], PREFAB 4.0 [7] and BAliBASE 3.0 [18]. These are the databases of structure-based alignments for homologous families. We used the all 630 pairwise alignments obtained from the HOMSTRAD for pairwise alignment tests, and used the all 1031 multiple alignments obtained from this database for multiple alignment tests. We also used the all 1682 protein pairs obtained from the PREFAB 4.0 for pairwise alignment tests. The BAliBASE 3.0 contains 5 different reference sets of alignment for testing multiple sequence alignment methods. We used the BBS sets included in the references 1,2,3 and 5. The BBS sets are described as being trimmed to homologous regions.

In order to avoid using the same dataset for training and test, We estimated the transition quantity by using the superfamilies subset from the dataset SABmark, which is described in the section "Estimation of the Transition Quantity". We also used this dataset for optimizing the parameters *w*_open_, *w*_extend_, ϵ. Consequently, MTRAP uses the followings for parameter values: *w*_open _= *f*_*s *_(-11), *w*_extend _= *f*_*s*_(-0.3) and *ε *= 0.775.

Alignment accuracy was calculated with the *Q *(quality) score [7]. The *Q *score is defined as the ratio of the number of correctly aligned residue pairs in the test alignment (i.e., the alignment obtained by a specified algorithm such as MTRAP, Needle, etc.) to the total number of aligned residue pairs in the reference alignment. When all pairs are correctly aligned, the score have a maximum value 1, and when no-pairs are aligned the score have a minimum value 0. This score has previously been called the SPS (Sum of Pairs Score) [29] or the developer score [30]. Let us redefined this score in our notations. Let *A*_*i *_(*i *= 1, i..., *N*) indicates the *i*th sequence of the reference alignment with length *L*, and let *a*_*ik *_∊ Ω* indicates the *k*th symbol in the sequence *A*_*i*_. When *a*_*ik *_≠ *, it is important to find the number of the site in the test sequence corresponding to the symbol *a*_*ik*_, whose numbers are denoted by *n*_*ik*_. When *a*_*ik *_= *, put *n*_*ik *_= 0 (*i *= 1, *..., N*). Then the *Q *score is given as

### Implementation

The MTRAP algorithm is implemented as a C++ program. The program has been tested in several types of Linux machines including 32-bit ×86 platform and also has been tested on Mac OSX snow leopard (64-bit). The program has a number of command-line options, e.g., the option setting the value of a parameter such that , , *ε*, *w*_open_, *w*_extend_, and the option controlling the output format. The program accepts only multiple-fasta format as an input format.

## Results and Discussion

### Performance evaluation of pairwise alignment

We compared MTRAP with nine different alignment methods including the modified TCoffee by using all 1682 protein pairs of PREFAB 4.0 and all 630 protein pairs of HOMSTRAD. We used GONNET250 matrix with the MTRAP. The similarity between the test alignment (sequence alignment by each method) and the reference alignment (obtained from PREFAB 4.0 or HOMSTRAD) was measured with the Q score. Suppose as usual that the reference alignment is the optimal alignment, the results of PREFAB 4.0 (Table 1) and those of HOMSTRAD (Table 2) indicate that our method works well compared with other methods. Our method achieves the highest ranking compared with all other methods except only one range 30-45%. Especially for the identity range 0-15%, MTRAP is 4 ~ 5% accurate than the 2nd ranking method. For the identity range 30-45%, Probcons and Probalign perform slightly better (~ 1%).

**Table 1:** Average Q scores on the PREFAB 4.0 database.

Method	PREFAB 4.0				
	
	0-15%(212)	15-30%(458)	30-45%(74)	All(1682)	CPU
MTRAP^a^	**0.248**	**0.674**	0.877	**0.615**	120
MAFFT	0.170	0.671	0.860	0.568	200
DIALIGN^b^	0.133	0.556	0.814	0.518	100
MUSCLE	0.205	0.632	0.867	0.581	35
ClustalW2	0.199	0.644	0.859	0.586	70
Probcons	0.204	0.647	0.875	0.590	120
Probalign	0.195	0.654	**0.887**	0.593	100
TCoffee	0.198	0.642	0.872	0.585	180
TCoffee-Lu/Sze	0.198	0.647	0.874	0.588	270

The average Q scores of four testing datasets with different identity ranges on PREFAB 4.0 are shown. The number in parentheses denotes the number of alignments in each sequence identity range. For each sequence identity range, the best scores are in bold. CPU is the total computing time for all alignments in seconds.

^a^MTRAP uses GONNET250 substitution matrix.

^b^DIALIGN reported critical errors for some testing data. Therefore, the scores of DIALIGN were calculated by the partial testing data.

**Table 2:** Average Q scores on the HOMSTRAD database (Pairwise only).

Method	HOMSTRAD (Pairwise only)				
	
	0-15%(25)	15-30%(207)	30-45%(173)	All(630)	CPU
MTRAP^a^	**0.412**	**0.659**	0.879	**0.819**	45
MAFFT	0.309	0.610	0.863	0.796	60
DIALIGN^b^	0.216	0.546	0.825	0.760	35
MUSCLE	0.337	0.625	0.868	0.802	15
ClustalW2	0.313	0.619	0.867	0.800	25
Probcons	0.344	0.650	0.884	0.816	50
Probalign	0.325	0.649	**0.886**	0.818	40
TCoffee	0.341	0.634	0.872	0.809	70
TCoffee-Lu/Sze	0.347	0.649	0.879	0.815	100

The average Q scores of four testing datasets with different identity ranges on HOMSTRAD are shown. The notations are the same as Table 1.

### Performance evaluations using other substitution matrices

**Figure 3:**
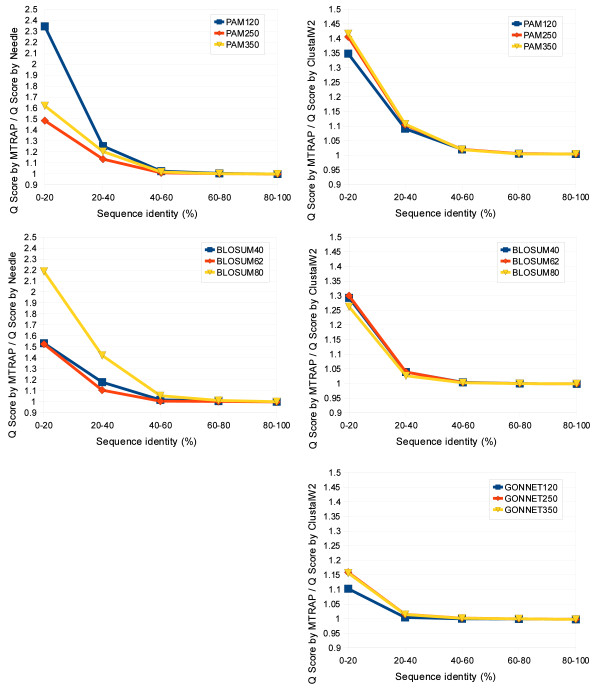
**The ratios of the average Q scores on the PREFAB 4.0 database**. The upper two figures show the ratio of the average Q score by MTRAP to that by Needle and the ratio of ours to that by ClustalW2, both for PAM substitution matrix. The middle two figures show the ratios for BLOSUM substitution matrix. The last figure shows the ratio for GONNET substitution matrix.

We did the performance evaluations using three different substitution matrix series: PAM, BLOSUM and GONNET, with HOMSTRAD and PREFAB 4.0, whose results are shown in Table 3 and Figure 3, respectively. We compared MTRAP with two typical global alignment programs, Needle and ClustalW2, which can use various substitution matrices. We used all 630 protein pairs of HOMSTRAD and all 1682 protein pairs of PREFAB 4.0. The similarity between the test alignment and the reference alignment was measured with the Q score.

**Table 3:** Average Q scores in pairwise alignment tests with typical substitution matrices.

Matrix	HOMSTRAD (Pairwise only)			
	
Method	0-15% (25)	15-30% (207)	30-45% (173)	All (630)
PAM250				
MTRAP	**0.421**	**0.655**	**0.874**	**0.817**
Needle	0.226	0.548	0.837	0.763
ClustalW2	0.234	0.528	0.817	0.747

BLOSUM62				
MTRAP	**0.410**	**0.653**	**0.878**	**0.817**
Needle	0.223	0.556	0.843	0.768
ClustalW2	0.276	0.585	0.861	0.784

GONNET250*				
MTRAP	**0.412**	**0.659**	**0.879**	**0.819**
ClustalW2	0.313	0.619	0.867	0.800

The average Q scores of four testing datasets with different identity ranges on HOMSTRAD are shown. The number in parentheses denotes the number of alignments in each sequence identity range. For each sequence identity range, the best scores in each substitution matrix are in bold.

*Needle does not support GONNET matrix.

For every typical substitution matrix (i.e., PAM250, BLOSUM62 and GONNET250), MTRAP has more than 80% accuracy (e.g., 0.817 with PAM250 and BLOSUM62), whereas Needle and ClustalW2 have less than 80% accuracy (e.g., Needle has 0.763 with PAM250 and 0.768 with BLOSUM62) (Table 3). Moreover, it is important to notice that for two sequences with less than 30% sequence identity, our method improves the alignment accuracy significantly. For instance, MTRAP with PAM250 matrix has 0.421 for 0-15% sequence identity and 0.655 for 15-30% sequence identity, and ClustalW2 with PAM250 matrix has 0.234 for 0-15% sequence identity and 0.528 for 15-30% sequence identity, respectively.

Figure 3 shows the results with another database PREFAB 4.0 that are the ratios of the average Q scores for each identity range. For all substitution matrices, these three programs show almost the same alignment accuracy when the sequence identity is more than 60%, whereas the ratio clearly shows that MTRAP has higher accuracy than other programs in decreasing the sequence identity within 0-60%. For instance, MTRAP and Needle with PAM120 have 0.356 and 0.152 for 0-20% sequence identity, and those with BLOSUM80 have 0.363 and 0.166, respectively. For alignments with sequence identity 0-20%, the average Q score of MTRAP is 1.5-2.3 times more accurate than that of Needle. Moreover, MTRAP outperforms ClustalW2 at the same range by 1.4, 1.3 and 1.1-1.2 times for PAM, BLOSUM and GONNET series, respectively.

### Performance of MTRAP algorithm for multiple alignment

We modified the TCoffee by means of our MTRAP algorithm. Table 4 and Table 5 show the accuracy of the modified TCoffee (TCoffee-MTRAP) compared with other methods including the original TCoffee with HOMSTRAD and BAliBASE 3.0. For all testing datasets, TCoffee-MTRAP shows the performance increase over the original TCoffee. Especially for the identity range 0-15%, TCoffee-MTRAP is 8.0% more accurate than the original TCoffee with HOMSTRAD, whereas the TCoffee modified by the Lu/Sze algorithm (TCoffee-Lu/Sze) is 0.7% more accurate than the original (Table 4). Also for V1 (i.e., the sequence identity is less than 20%) of the reference 1, TCoffee-MTRAP is 6.0% more accurate than the original TCoffee on BAliBASE 3.0, whereas TCoffee-Lu/Sze is 0.3% more accurate than the original (Table 5). In some domains, the two methods Probcons and Probalign, both of which are based on the probabilistic consistency strategy, are more accurate than TCoffee-MTRAP. Note that these two methods use the parameter values estimated from the BAliBASE 2.0 database.

**Table 4:** Average Q scores on the HOMSTRAD database.

Method	HOMSTRAD			
	
	0-15%(32)	15-30%(325)	30-45%(331)	All(1031)
TCoffee-MTRAP	**0.395**	**0.666**	**0.868**	**0.819**
TCoffee-Lu/Sze	0.322	0.648	**0.868**	0.813

TCoffee	0.315	0.642	0.864	0.809
MAFFT	0.288	0.632	0.858	0.803
DIALIGN*	0.203	0.559	0.811	0.761
MUSCLE	**0.333**	0.643	0.860	0.809
ClustalW2	0.313	0.628	0.855	0.815
Probcons	0.329	0.666	0.873	0.820
Probalign	0.310	**0.670**	**0.877**	**0.824**

The average Q scores of four testing datasets with different identity ranges on HOMSTRAD are shown. For each sequence identity range, the better scores of the TCoffee modified by Lu/Sze algorithm (TCoffee-Lu/Sze) and the TCoffee modified by MTRAP algorithm (TCoffee-MTRAP) are in bold. The best scores of the other methods are also in bold.

*DIALIGN reported critical errors for some testing data. Therefore, the scores of DIALIGN were calculated by the partial testing data.

**Table 5:** Average Q scores on the BAliBASE 3.0 database.

Method	Reference 1		Reference 2	Reference 3	Reference 5
	Equidistant Sequences		Family with	Divergent	Large
	V1:0-20%ID	V2:20-40%ID	"Orphans"	subfamilies	Insertions
TCoffee-MTRAP	**0.752**	**0.943**	**0.947**	**0.873**	**0.892**
TCoffee-Lu/Sze	0.695	0.937	0.939	0.851	0.879

TCoffee	0.692	0.936	0.940	0.849	0.874
MAFFT	0.722	0.901	0.945	0.864	0.900
DIALIGN*	0.566	0.860	0.883	0.766	0.861
MUSCLE	0.743	0.931	0.941	0.870	0.872
ClustalW2	0.654	0.903	0.922	0.821	0.805
Probcons	**0.811**	**0.951**	**0.957**	**0.905**	**0.909**
Probalign	0.728	0.947	0.945	0.876	0.893

The average Q scores of five reference BBS sets (described as being trimmed to homologous regions) on BAliBASE 3.0 are shown. For each reference, the better scores of the TCoffee modified by Lu/Sze algorithm (TCoffee-Lu/Sze) and the TCoffee modified by MTRAP algorithm (TCoffee-MTRAP) are in bold. The best scores of the other methods are also in bold. ID means a sequence identity of the reference alignment.

*DIALIGN reported critical errors for some testing data. Therefore, the scores of DIALIGN were calculated by the partial testing data.

## Conclusions

MTRAP is a global alignment method that is based on a new metric. The metric is determined by an adjusted substitution matrix and a transition probability-based matrix between two consecutive pairs of residues including gap-residue derived from structure-based alignments.

We indicated here that our approach, which takes into account an intersite correlation on the sequences, leads to a significant increase in the alignment accuracy, especially, for the low identity range. We also indicated that the MTRAP improves the alignment accuracy for any substitution matrices. Moreover, we confirmed that our algorithm works well together with a consistency based progressive approach for constructing multiple alignment.

However, the methods Probcons and Probalign were more accurate than TCoffee-MTRAP in some multiple alignment tests. The probabilistic consistency strategy is an improved consistency strategy of TCoffee. Therefore, combining MTRAP pairwise algorithm with the probabilistic consistency strategy will generate more high quality multiple alignments. We will examine this fact in a separate paper.

MTRAP has the similar calculation cost with other pairwise methods. That is, MTRAP has *O *(*mn*) calculation order for two input sequences with length *m *and *n*. Our CPU time shown in the Tables 1, 2 are almost the same as others.

Pairwise sequence alignment is among the most important technique to perform biological sequence analysis, and is fundamental to other applications in bioinformatics. Any multiple sequence alignment that is gradually built up by aligning pairwise sequences is essentially based on high-quality pairwise sequence alignments. By modifying common multiple alignment method based on our algorithm as shown in this paper, the accuracy was improved significantly. Moreover, we think that our technique is applicable to not only global alignment, but also some others such as, local homology search and motif-finding, which will be our future works.

## Availability and requirements

Project name: MTRAP

Project home page: http://www.rs.noda.tus.ac.jp/%7Eohya-m/

Operating systems: Linux, UNIX

Programming languages: C++

License: BSD license

## Authors' contributions

We three (TH, KS, MO) discussed all fundamental parts together. In details, mathematical idea mainly comes from MO and TH did mathematical algorithm. Moreover, TH and KS made computer algorithm and did computer alignment by means of this algorithm. All authors have read and approved the final manuscript.

## References

[B1] Pearson W Lipman D Improved tools for biological sequence comparison Proceedings of the National Academy of Sciences 1988 85 8 2444 2448 10.1073/pnas.85.8.2444 PMC280013 3162770

[B2] Altschul S Gish W Miller W Myers E Lipman D Basic local alignment search tool Journal of molecular biology 1990 215 3 403 410 2231712 10.1016/S0022-2836(05)80360-2

[B3] Thompson JD Higgins DG Gibson TJ CLUSTAL W: improving the sensitivity of progressive multiple sequence alignment through sequence weighting, position-specific gap penalties and weight matrix choice Nucleic Acids Res 1994 22 4673 4680 10.1093/nar/22.22.4673 7984417 PMC308517

[B4] Morgenstern B DIALIGN 2: improvement of the segment-to-segment approach to multiple sequence alignment Bioinformatics 1999 15 211 218 10.1093/bioinformatics/15.3.211 10222408

[B5] Notredame C Higgins DG Heringa J T-Coffee: A novel method for fast and accurate multiple sequence alignment J Mol Biol 2000 302 205 217 10.1006/jmbi.2000.4042 10964570

[B6] Katoh K Misawa K Kuma K Miyata T MAFFT: a novel method for rapid multiple sequence alignment based on fast Fourier transform Nucleic Acids Res 2002 30 3059 3066 10.1093/nar/gkf436 12136088 PMC135756

[B7] Edgar RC MUSCLE: multiple sequence alignment with high accuracy and high throughput Nucleic Acids Res 2004 32 1792 1797 10.1093/nar/gkh340 15034147 PMC390337

[B8] Do C Mahabhashyam M Brudno M Batzoglou S ProbCons: probabilistic consistency-based multiple sequence alignment Genome Research 2005 15 2 330 10.1101/gr.2821705 15687296 PMC546535

[B9] Roshan U Livesay D Probalign: multiple sequence alignment using partition function posterior probabilities Bioinformatics 2006 22 22 2715 10.1093/bioinformatics/btl472 16954142

[B10] Feng D Doolittle R Progressive sequence alignment as a prerequisite to correct phylogenetic trees Journal of Molecular Evolution 1987 25 4 351 360 10.1007/BF02603120 3118049

[B11] Blackshields G Wallace I Larkin M Higgins D Analysis and comparison of benchmarks for multiple sequence alignment In Silico Biology 2006 6 4 321 339 16922695

[B12] Wang K Samudrala R Incorporating background frequency improves entropy-based residue conservation measures BMC bioinformatics 2006 7 385 10.1186/1471-2105-7-385 16916457 PMC1562451

[B13] Gotoh O Consistency of optimal sequence alignments Bulletin of Mathematical Biology 1990 52 4 509 525 1697773 10.1007/BF02462264

[B14] Anfinsen CB Principles that govern the folding of protein chains Science 1973 181 223 230 10.1126/science.181.4096.223 4124164

[B15] Gonnet G Cohen M Benner S Analysis of amino acid substitution during divergent evolution: the 400 by 400 dipeptide substitution matrix Biochemical and Biophysical Research Communications 1994 199 489 489 10.1006/bbrc.1994.1255 8135790

[B16] Crooks G Green R Brenner S Pairwise alignment incorporating dipeptide covariation Bioinformatics 2005 21 19 3704 10.1093/bioinformatics/bti616 16123116

[B17] Lu Y Sze S Improving accuracy of multiple sequence alignment algorithms based on alignment of neighboring residues Nucleic Acids Research 2009 37 2 463 10.1093/nar/gkn945 19056820 PMC2632924

[B18] Thompson JD Koehl P Ripp R Poch O BAliBASE 3.0: latest developments of the multiple sequence alignment benchmark Proteins 2005 61 127 136 10.1002/prot.20527 16044462

[B19] Altschul S Amino acid substitution matrices from an information theoretic perspective J Mol Biol 1991 219 555 565 10.1016/0022-2836(91)90193-A 2051488 PMC7130686

[B20] Van Walle I Lasters I Wyns L SABmark - a benchmark for sequence alignment that covers the entire known fold space Bioinformatics 2005 21 7 1267 10.1093/bioinformatics/bth493 15333456

[B21] Murzin A Brenner S Hubbard T Chothia C SCOP: a structural classification of proteins database for the investigation of sequences and structures Journal of molecular biology 1995 247 4 536 540 7723011 10.1006/jmbi.1995.0159

[B22] Lipman D Altschul S Kececioglu J A tool for multiple sequence alignment Proceedings of the National Academy of Sciences 1989 86 12 4412 4415 10.1073/pnas.86.12.4412 PMC287279 2734293

[B23] Ohya M Uesaka Y Amino acid sequences and DP matching:a new method of alignment Information Sciences 1992 63 139 151 10.1016/0020-0255(92)90065-G

[B24] Needleman SB Wunsch CD A general method applicable to the search for similarities in the amino acid sequence of two proteins Journal of Molecular Biology 1970 48 443 453 10.1016/0022-2836(70)90057-4 5420325

[B25] Rice P Longden I Bleasby A EMBOSS: the European Molecular Biology Open Software Suite Trends Genet 2000 16 276 277 10.1016/S0168-9525(00)02024-2 10827456

[B26] Larkin MA Blackshields G Brown NP Chenna R McGettigan PA McWilliam H Valentin F Wallace IM Wilm A Lopez R Thompson JD Gibson TJ Higgins DG Clustal W and Clustal X version 2.0 Bioinformatics 2007 23 2947 2948 10.1093/bioinformatics/btm404 17846036

[B27] Mizuguchi K Deane CM Blundell TL Overington JP HOMSTRAD: a database of protein structure alignments for homologous families Protein Sci 1998 7 2469 2471 10.1002/pro.5560071126 9828015 PMC2143859

[B28] Stebbings L Mizuguchi K HOMSTRAD: recent developments of the homologous protein structure alignment database Nucleic acids research 2004 32 Database D203 10.1093/nar/gkh027 14681395 PMC308761

[B29] Thompson J Plewniak F Poch O A comprehensive comparison of multiple sequence alignment programs Nucleic Acids Res 1999 27 13 2682 2690 10.1093/nar/27.13.2682 10373585 PMC148477

[B30] Sauder J Arthur J Dunbrack R Jr Large-scale comparison of protein sequence alignment algorithms with structure alignments Proteins Structure Function and Genetics 2000 40 6 22 10.1002/(SICI)1097-0134(20000701)40:1<6::AID-PROT30>3.0.CO;2-7 10813826

